# Polymorph II Cellulose Nanocrystals Derived from Oil Palm Empty Fruit Bunches for High-Efficiency COD Removal in Industrial Wastewater

**DOI:** 10.3390/nano16060374

**Published:** 2026-03-20

**Authors:** Jemina Pomalaya-Velasco, Yéssica Bendezú-Roca, Yamerson Canchanya-Huaman, Juan A. Ramos-Guivar

**Affiliations:** 1 Laboratorio de No Metálicos, Facultad de Ingeniería Química, Universidad Nacional del Centro del Perú (UNCP), Av. Mariscal Ramón Castilla N° 3909, El Tambo, Huancayo 12000, Peru; 2 Grupo de Investigación de Nanotecnología Aplicada para Biorremediación Ambiental, Energía, Biomedicina y Agricultura (NANOTECH), Facultad de Ciencias Físicas, Universidad Nacional Mayor de San Marcos, Av. Venezuela Cdra 34 S/N, Ciudad Universitaria, Lima 15081, Peru

**Keywords:** cellulose II nanocrystals, oil palm empty fruit bunch, chemical oxygen demand (COD) removal, industrial wastewater treatment, biomass-derived material, polymorph transformation, Box–Behnken design, sustainable water remediation

## Abstract

This study reports the valorization of oil palm empty fruit bunches into cellulose nanocrystals (CNCs) for the removal of the chemical oxygen demand (COD) from industrial wastewater generated by the same processing sector. Cellulose I_β_ was first isolated through sequential bleaching, delignification, and mercerization, and two hydrolysis routes were evaluated to obtain CNCs: a concentrated acid route (60% *v*/*v* H_2_SO_4_, 50 °C, 60 min) for CNCs-1 and a low-acid, long-duration route (1% *v*/*v* H_2_SO_4_, 80 °C, 12 h) for CNCs-2. Rietveld refinement of the X-ray diffractograms confirmed the polymorphic transition, assigning cellulose I_β_ to the intermediate materials and cellulose II to the CNC samples, with crystallite sizes of 4.99 nm for CNCs-1 and 5.43 nm for CNCs-2. Attenuated Total Reflectance–Fourier Transform Infrared (ATR-FTIR) spectroscopy analysis showed the progressive removal of lignin and hemicellulose and supported the cellulose I_β_ to II transition through changes in hydroxyl bonding and crystallinity-related bands. Preliminary adsorption tests showed better COD removal with CNCs-2, which were therefore selected for optimization using a Box–Behnken design with the adsorbent mass, pH, and contact time as variables. The quadratic model was significant (R^2^ = 0.9675; predicted R^2^ = 0.8908), and the maximum COD removal reached 91.47%, decreasing the COD concentration from 2459.0 to 209.85 mg L^−1^ under the optimum conditions of 0.09 g CNCs-2, pH 3, and 20 min. These results highlight cellulose II nanocrystals derived from oil palm waste as a promising and scalable adsorbent for industrial wastewater treatment.

## 1. Introduction

Rapid industrial and urban development has led to the discharge of large volumes of industrial wastewater, increasing water pollution. The oil-seed industry is one of the fastest-growing industrial sectors in the world. Over the last decade, the palm oil processing industry has experienced progressive growth due to high demand, reaching 78.76 million tons in 2024, with projected growth of 2.8% by 2034 [1]. This has led to increased production capacity and, consequently, the generation of considerable volumes of industrial wastewater with high concentrations of organic compounds [2]. Approximately 1.57 L of water is used to obtain 1 L of crude palm oil; on average, 75% of the water used in the extraction industry is discharged as wastewater [3]. This wastewater has a high organic load, which is reflected in the COD, a parameter used to determine the amount of organic matter susceptible to chemical oxidation present in the water [4]. The discharge of wastewater containing concentrations exceeding 1500 mg L^−1^ of O_2_ (maximum permissible limit (MPL), Ministry of Environment and Sustainable Development, Colombia 2015) [5] poses a threat that can lead to a decrease in dissolved oxygen (DO) in receiving water bodies, affecting the survival of aquatic organisms and disrupting the balance of ecosystems. Furthermore, the accumulation of organic matter can increase water turbidity and reduce light penetration, limiting photosynthetic processes and affecting the ecological dynamics of the aquatic system [6], representing a significant environmental risk. Therefore, there is growing interest in developing efficient and sustainable treatment strategies to reduce the organic load present in this wastewater and mitigate the impact on water resources.

Several wastewater remediation techniques using organic substances have been developed, including biological treatments [7], coagulation and flocculation [8,9], advanced oxidation [10,11], and photocatalysis [12,13]. While these methods are efficient, they have limitations, such as the generation of secondary waste, high energy consumption, and the requirement for chemical reagents and expensive equipment. Nanotechnology has emerged as a promising solution for COD removal; for example, titanium oxide nanoparticles used in photocatalytic processes have reported COD removal efficiencies of between 60% and 70% [14]. Consequently, interest in developing materials from lignocellulosic waste has increased, making it an important source for producing high-value-added materials, due in part to their abundance, easy availability, and biodegradability [15]. The oilseed industry produces abundant quantities of lignocellulosic waste, which is only partially utilized, causing additional environmental impacts. However, these biodegradable wastes represent an opportunity for obtaining materials applicable in environmental remediation.

Among them, the African oil palm (*Elaeis guineensis*), widely used for palm oil production, generates empty fruit bunches as its main byproduct, which are largely discarded or valorized as compost [16]. These oil palm empty fruit bunches, a lignocellulosic residue, typically contain approximately 32.8% cellulose, 13.74% hemicellulose, and 30.12% lignin [17]. Efficient separation of these components remains one of the main challenges for the effective utilization of this biomass.

Cellulose is the most abundant organic polymer in nature [18] and has been classified as a non-toxic and fully biodegradable material that does not generate adverse effects on the environment or humans [19]. Intra- and intermolecular hydrogen bonding between glucopyranose units in cellulose gives rise to four distinct crystalline forms (I–IV). Native cellulose occurs in two polymorphs, the triclinic I_α_ and the monoclinic I_β_, which are mainly found in bacteria and plants, respectively [20], while chemical treatments allow conversion to cellulose II, III, and IV [21]. Cellulose II, with its monoclinic crystalline structure, antiparallel lamellar packing, and higher intramolecular hydrogen bonding, exhibits improved mechanical performance, including higher fracture strain compared to cellulose I, making it more suitable for industrial applications [22]. To achieve greater removal of lignin and hemicellulose, bleaching is carried out using oxidizing agents such as sodium chlorite (NaClO_2_) [23]. Subsequently, alkaline treatment converts cellulose I into the cellulose II polymorph, while also promoting the residual removal of hemicellulose and lignin [24]. The removal of lignin and hemicellulose to obtain cellulose II from a lignocellulosic source can be achieved using an alkaline solution [24,25,26]. One of the most commonly used methods to induce structural changes is mercerization [27,28]. Polymorph II provides better performance than I, particularly by improving the mechanical properties and structural and thermal stability, as well as mechanical strength [29,30].

Structurally, cellulose is characterized by the coexistence of highly ordered crystalline regions and less ordered amorphous domains [31]. To obtain CNCs from purified cellulose sources, acid hydrolysis is the most widely used method. Commonly employed acids include sulfuric, phosphoric, and hydrochloric acids, as well as their mixtures [24]. Sulfuric acid is particularly attractive as it produces CNC suspensions with good stability in aqueous media due to the introduction of negatively charged sulfate groups on the surface [32,33,34]. At the nanoscale, these structures offer excellent mechanical properties and hydrophilicity, as well as affinities for different classes of pollutants [35], making them a promising alternative.

The production of CNCs from abundant empty fruit bunches offers significant potential for cost-effective and large-scale wastewater treatment. Various cellulosic sources, such as sugarcane bagasse, cotton, and rice husk [36], have been explored; however, their availability is often limited by seasonal and geographical factors. In contrast, empty fruit bunch residues generated during oil palm processing are produced daily and are readily available. Limited research has been reported on the use of biomass-derived CNCs for the removal of COD from wastewater. Among the reported materials, nanocellulose acetate, derived from dried leaves of *Combretum indicum*, has shown removal efficiencies of approximately 86% [4]. Similarly, nanocellulose obtained from oil palm residues has demonstrated removal efficiencies of around 70% [35], highlighting the potential of lignocellulosic materials as sustainable materials for wastewater treatment. The utilization of these residues for wastewater treatment not only contributes to efficient COD reduction but also promotes the valorization of lignocellulosic waste. However, studies employing residues generated within the same industry for the treatment of its own wastewater remain limited. Therefore, the use of lignocellulosic residues produced in the oil palm processing industry for the removal of COD from its own wastewater represents a promising and sustainable strategy, enabling the simultaneous management of industrial residues and wastewater.

In this context, the present study aimed to synthesize CNCs from empty fibers of oil palm fruit bunches—waste generated from a palm oil production plant—for their subsequent application in COD removal from wastewater generated by the same production process. CNCs were extracted from *Elaeis guineensis* by removing lignin and hemicellulose using NaClO_2_, sodium sulfite (Na_2_SO_3_), and NaOH, following the procedure of [24], with modified parameters and operating conditions. This was followed by the selective removal of the amorphous cellulose phase using two different concentrations of H_2_SO_4_. The CNCs obtained were characterized by X-ray diffraction (XRD) to determine the polymorph type, crystallite size, and degree of crystallinity (DOC), while ATR-FTIR spectroscopy was employed to analyze intra- and intermolecular hydrogen bonding, differentiate cellulose I and II, and confirm the removal of lignin and hemicellulose. The resulting CNCs were used for COD removal from palm oil plant wastewater, optimizing the process through a Box–Behnken design (BBD) that controlled for the CNCs mass, treatment pH, and contact time.

## 2. Materials and Methods

### 2.1. Chemical Reagents

Extra-pure glacial acetic acid (CH_3_COOH) and sodium chlorite (NaClO_2_, 80%) were obtained from KERM S&D (Cuarte de Huerva, Zaragoza, Spain); sulfuric acid (H_2_SO_4_, 96%), sodium hydroxide pellets (NaOH, 98%, anhydrous), and ethanol (C_2_H_5_OH) were supplied by Scharlab S.L. (Sentmenat, Barcelona, Spain); and sodium sulfite (Na_2_SO_3_, 98%) was provided by HiMedia Laboratories (Thane (West), Maharashtra, India).

The wastewater used for the COD removal experiments was collected according to Peruvian Technical Standard 214.005-2016 [37] and ISO 5667-10:2012 [38] from a palm oil production plant located in the district of Neshuya, Ucayali, Peru. Likewise, the empty palm bunches used to synthesize CNCs were collected from waste from the same oil palm production process. The process for extracting cellulose from fibers of empty oil palm fruit bunches was adapted from a previously reported method [39].

### 2.2. Preparation of CNCs

#### 2.2.1. Cellulose Isolation from Empty Bunches of Oil Palm Fruit

The fiber from empty bunches of oil palm fruit (SCP) was bleached with a NaCl_2_O (0.7% *w*/*v*) solution in a 1:50 *w*/*v* ratio. The pH was adjusted to 5 with CH_3_COOH (10% *v*/*v*). Then, the material was homogenized using a magnetic stirrer at 300 rpm, increasing the temperature to 70 °C and keeping it constant for 2 h. The procedure was repeated four times until the fiber acquired a white color. The suspension was cooled to room temperature (RT) and then vacuum-filtered and washed with distilled water until pH 7 was reached. It was then dried in an oven at 40 °C for 24 h. The bleached fiber (SCB) was suspended in Na_2_SO_3_ (5% *w*/*v*) at 70 °C for 1 h, at a ratio of 1:40, and then vacuum-filtered and dried at 40 °C for 12 h. The delignified fiber (SCS) was suspended in NaOH (17.5% *w*/*v*) at 25 °C for 2 h, vacuum-filtered, and washed with distilled water and C_2_H_5_OH until pH 7 was reached. It was then dried at 40 °C for 12 h, thus obtaining cellulose (SC).

#### 2.2.2. Synthesis of CNCs

CNCs were obtained by acid hydrolysis, following two synthesis routes.

Route 1:

CNCs-1 were obtained from cellulose by adding H_2_SO_4_ (60% *v*/*v*) dropwise under stirring at 350 rpm, in a 1:20 *w*/*v* ratio, maintaining the temperature below 30 °C for 5 min to allow complete acid transfer. The temperature was then raised to 50 °C for 60 min. The hydrolysis process was quenched by adding twice the volume of distilled water relative to the acid solution. The mixture was washed with copious amounts of distilled water until pH 4 was reached, and it was then neutralized with NaOH (0.5% *w*/*v*), added dropwise until a neutral pH was reached, with stirring for 10 min. Finally, the mixture was centrifuged at 6000 rpm for 15 min to separate the precipitate from the liquid and subsequently sonicated for 15 min.

Route 2:

CNCs-2 were extracted from cellulose by adding H_2_SO_4_ (1% *v*/*v*) under stirring at 350 rpm in a closed beaker, in a 1:35 *w*/*v* ratio, maintaining the temperature below 80 °C for 12 h. The hydrolytic mixture was cooled to RT. Subsequently, it was washed with distilled water followed by C_2_H_5_OH. This process was repeated until the suspension reached pH 7. Finally, it was dried at 40 °C for 12 h.

### 2.3. Selection of CNCs for COD Removal Experiments

Preliminary experiments were conducted to select the CNCs used in the COD removal tests. In each experiment, 1.5 g of the respective material (CNCs-1 or CNCs-2) was placed in two beakers and brought into contact with 50 mL of wastewater from a tannery, which had an initial COD concentration of 876 mg L^−1^. The mixture was stirred at 250 rpm for 90 min at its natural pH of 7.10. The solutions were then filtered, and their final concentrations were determined. CNCs-1 exhibited a final COD concentration of 600 mg L^−1^, while that of CNCs-2 reached 360 mg L^−1^, corresponding to COD removal percentages of 31.5% and 58.9%, respectively. Therefore, CNCs-2, which exhibited the highest COD removal capacity, obtained via method 2, were selected for subsequent treatments.

### 2.4. Characterization

#### 2.4.1. ATR-FTIR Experimental Conditions

ATR-FTIR spectroscopy analysis of all samples was performed with a spectral resolution of 4 cm^−1^, in a spectral range of 4000 to 650 cm^−1^, using a Lyza 7000 spectrometer (Anton Paar GmbH, Graz, Austria). The spectra were recorded in the 4000–650 cm^−1^ range because this interval includes the main diagnostic vibrations used here to assess cellulose purification and polymorphic transitions, whereas the region below 650 cm^−1^ did not provide additional information relevant to the present structural interpretation.

#### 2.4.2. XRD Experimental Conditions

The XRD data were collected using a Rigaku diffractometer (Tokyo, Japan), operating with CuK_α_ radiation (1.5406 Å) at 50 kV and 100 mA. The experimental datasets used for refinement corresponded to the measured range 2*θ* = 5–40° (step size: 0.02° and scan rate: 2 s/step) for cellulose I_β_ and II. Rietveld refinements were performed with FullProf Suite (version January 2021) using the Thompson–Cox–Hastings (TCH) pseudo-Voigt peak-shape function with axial divergence asymmetry correction [40,41]. The crystallographic models were imported directly from the provided CIF files, where both models are monoclinic, of Laue class 2/m, with space group P2_1_. For cellulose II [42], cell parameters were a = 8.10 Å, b = 9.03 Å, c = 10.31 Å, α = β = 90°, γ = 117.10°; for cellulose I_β_ [43], a = 7.784 Å, b = 8.201 Å, c = 10.380 Å, α = β = 90°, γ = 96.55°, as an initial matching candidate. The crystallite size was calculated using the spherical harmonic approach implemented in FullProf [44].

*Crystallinity analysis.* The crystallinity of the extracted cellulose and prepared CNCs was analyzed by using X-ray diffractograms. The degree of crystallinity (*DOC*) was calculated from the integrated peak areas using Equation (1) [45]:

(1) $$DOC\left(\%\right)=\frac{A_{crystalline}}{A_{crystalline}+A_{amorphous}}\times 100$$

where *A*_crystalline_ = area of crystalline peaks and *A*_amorphous_ = area of non-crystalline peaks.

The primary objective of the characterization was to confirm the chemical purification of cellulose and the polymorphic transition to cellulose II nanocrystals. X-ray diffraction with Rietveld refinement provides the definitive identification of cellulose polymorphs and crystallographic parameters, while ATR-FTIR spectroscopy confirms the removal of lignin and hemicellulose and changes in hydrogen bonding. Additional techniques such as electron microscopy, BET surface area analysis, zeta potential studies, or thermal analysis, although useful in obtaining complementary information, were not essential to establish polymorphic identity or the chemical transformations addressed in this study and were therefore considered beyond its scope.

### 2.5. COD Removal Studies with CNCs

#### 2.5.1. Experimental Design

Once the factors with the greatest influence on the process were selected, the BBD was used with Design-Expert v13.0 to optimize COD removal. The study variables considered were the CNCs-2 mass (g), the pH of the treatment, and the contact time (min). Each variable had three levels, low (−1), medium (0), and high (+1), according to the coded values shown in Table 1. A set of 15 experiments was designed for this purpose. The experiments were performed in duplicate, and, after the statistical analysis of the results using ANOVA, the regression coefficients of the second-order model were estimated.

Subsequently, based on the combination of the results obtained, a quadratic polynomial equation was formulated that allowed the experimental data to be precisely fitted to the optimum point [46]. The response of the experimental system is described as follows:

(2) $$Y=\beta_{0}+\sum_{i=1}^{k}\beta_{i}x_{i}+\sum_{i=1}^{k}\beta_{ii}x_{i}^{2}+\sum_{i<j}^{k}\sum \beta_{ij}x_{i}x_{j}+\in$$

where Y represents the removal of COD, X_i_ and X_j_ represent the coded factors of the independent variables, β_0_ is the response value at the central level, β_i_ indicates the individual effect of each factor, β_ii_ the quadratic effect, β_ij_ the interaction of two factors and ∈ is the unknown error constant.

Because the COD represents the aggregate oxidizable organic content of a multicomponent wastewater, rather than a single adsorbate species, classical equilibrium isotherm models (e.g., Langmuir or Freundlich) and kinetic models developed for single-solute systems may not provide physically meaningful parameters for this system. Instead, the response surface methodology (RSM) using a Box–Behnken design was employed to evaluate the combined effects of process variables and their interactions, enabling the statistically robust optimization of COD removal under realistic treatment conditions.

#### 2.5.2. COD Removal Process

The removal of COD from wastewater collected from a palm oil production plant was evaluated with an initial concentration of 2459.0 mg L^−1^. The process consisted of using CNCs-2 of 0.03, 0.09, and 0.15 g, distributed into fifteen 100 mL flasks according to a Box–Behnken experimental design. These were placed in contact with 60 mL of wastewater. The pH was then adjusted and controlled with NaOH and H_2_SO_4_ (0.05 and 0.1 M) until pH values of 3, 5, and 7 were reached, with verification performed at regular intervals. The contact time was monitored at 20, 50, and 80 min. All experiments were conducted under constant stirring at 300 rpm and at RT. Subsequently, the sample was filtered to separate the solid and liquid phases. The liquid fraction was collected for the quantification of the final COD concentration by UV–vis spectrophotometry, using the closed-reflux, colorimetric method outlined in SMEWW-APHA-AWWA-WEF Part 5220 D, 24th Ed. 2023, Chemical Oxygen Demand [47]. The COD removal percentage was determined using Equation (3):

(3) $$D_{COD}(\%)=\frac{C_{0,COD}-C_{F,COD}}{C_{0,COD}}$$

where *D*_COD_ (%) is the COD removal percentage, and *C*_0,COD_ and *C*_F,COD_ are, respectively, the initial and final COD concentrations (mg L^−1^ O_2_).

## 3. Results and Discussion

### 3.1. ATR-FTIR Spectroscopy Analysis

**Figure 1:**
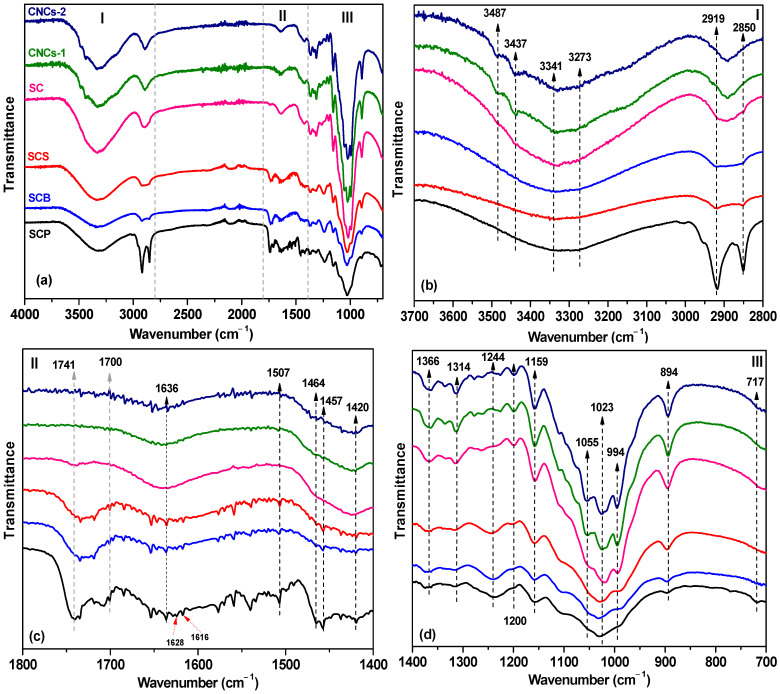
(**a**) ATR-FTIR spectra of SCP, SCB, SCS, SC, CNCs-1, and CNCs-2; (**b**) spectrum derived from (**a**) in the range of 3700 to 2800 cm^−1^ (Region I); (**c**) spectrum derived from (**a**) in the range of 1800 to 1400 cm^−1^ (Region II); (**d**) and spectrum derived from (**a**) in the range of 1400 to 700 cm^−1^ (Region III).

ATR-FTIR spectroscopy was used to identify the structural changes produced in the treatments applied for synthesis. The ATR-FTIR spectra of SCP, SCB, SCS, SC, CNCs-1, and CNCs-2 are shown in Figure 1a–d. The spectra show three main regions from 3700 to 2800 cm^−1^, 2800 to 1800 cm^−1^, and 1800 to 700 cm^−1^.

**Figure 2:**
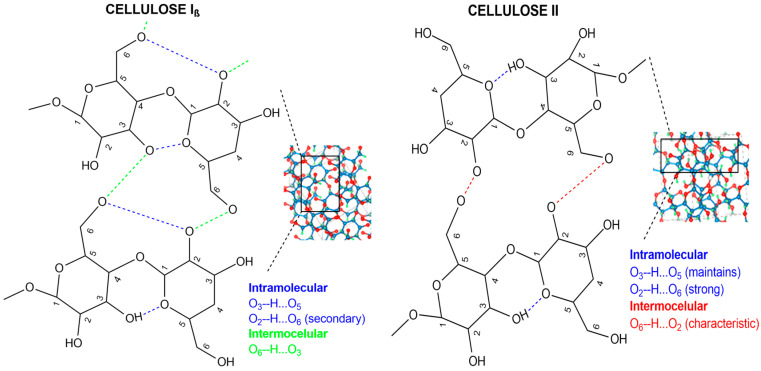
Chemical representation of intermolecular and intramolecular bonds in cellulose polymorphs (**left**) and II (**right**).

All spectra exhibited intense bands in the range of 3300 to 3500 cm^−1^ due to the stretching of the hydroxyl (-OH) group of cellulose [48,49]. The peak at 3341 cm^−1^, together with the weak band near 3273 cm^−1^, corresponds to the intramolecular O_3_′-H…O_5_′ and intermolecular O_6_′-H…O_3_′ bonds of polymorph I_β_, respectively [21,50]. After mercerization, the decrease in the intensity of these peaks and the appearance of two peaks at 3487 and 3437 cm^−1^ [21] evidenced the transition to cellulose polymorph II (see Figure 2). Likewise, the band at 1420 cm^−1^, known as the “crystallinity band”, corresponding to the flexural vibration of CH_2_ [51], showed a reduction in intensity, suggesting a decrease in crystallinity levels. In contrast, the increase in the band at 894 cm^−1^, considered as an “amorphous band” associated with the tensile vibration of C–O−C in β-(1 → 4) glycosidic bonds [25], is related to molecular reorganization in an antiparallel structure in polymorph II. These changes are significant because mercerization has been linked to the conversion of cellulose I to cellulose II [52] and to the emergence of amorphous regions with more accessible hydroxyl groups [53], while a reduction in crystalline order is associated with greater structural accessibility; both effects can facilitate interactions between the adsorbate and the surface and, therefore, contribute to the removal efficiency [54].

The transition from polymorphs I to II is also evidenced by the increase in the intensity of the band at 1366 cm^−1^ and the appearance of a band at 1314 cm^−1^ attributed to the CH_2_ vibrations of C2 and C6, respectively [55], confirming the structural reorganization. Changes in the peaks at 1055, 1023, and 994 cm^−1^ corresponding to the C–O bond in the glucopyranose ring [39,50,56] and the increase in intensity at 1159 cm^−1^, associated with the C-O-C stretching vibration [56], reflect the modification in the geometry of the glycosidic bond. In contrast, the band at 1200 cm^−1^ more clearly indicates the S=O vibration related to sulfate semi-ester groups [57].

Different characteristic peaks of lignin appear in the precursor material. The peaks at 2919 and 2850 cm^−1^ correspond to C–H stretching in methyl (-CH_3_) and methylene (-CH_2_) bonds [58], attributed to the aliphatic chains of suberin [59]. The peak at 1628 cm^−1^ is due to the C=C skeletal vibration of the benzene rings; the peak at 1616 cm^−1^ is due to the stretching of the C=O carboxyl groups [55,60]; that at 1507 cm^−1^ is due to the skeletal vibration of the C=C aromatic ring [61,62] and from the guaiacyl unit and C-H deformation in the methyl, methylene, and methoxyl groups [57]; and the peak near 1244 cm^−1^ is from the C-O stretching of the syringyl ring of lignin [58]. Likewise, the peaks at 1464 and 1457 cm^−1^ originate from the deformations of the methyl group and vibrations of the aromatic ring [55,61]. Hemicellulose groups, such as those indicated by the peaks at 1700 and 1741 cm^−1^, are related to C=O stretching belonging to the acetyl and uronic ester groups of hemicellulose or to carbonyl groups, including phenolic compounds such as *p*-coumaric acid [28,61,63]. After the pretreatments, these bands became less intense, especially after alkaline treatment, suggesting the removal of lignin and hemicellulose.

The progressive attenuation of lignin- and hemicellulose-associated bands, together with the increased prominence of cellulose-related absorptions after bleaching, delignification, and alkaline treatment, provides spectroscopic evidence of the substantial removal of non-cellulosic components. Although quantitative compositional analysis such as Klason lignin determination was not performed in this study, the chemical sequence employed is well established for the effective delignification of lignocellulosic biomass, and the ATR-FTIR/XRD results consistently support this interpretation.

The presence of cellulose is reflected at 1464 and 1457 cm^−1^, caused by deformations of the methyl group and vibrations of the aromatic ring [55,61]. The peak at 1636 cm^−1^ corresponds to the bending of H_2_O linked to (-OH) of cellulose, a product of deprotonation by NaOH [64]. The peak at 717 cm^−1^ for SCP is characteristic of CH_2_ rocking vibration in cellulose I_β_ (monoclinic).

### 3.2. X-Ray Diffraction and Rietveld Analysis

**Figure 3:**
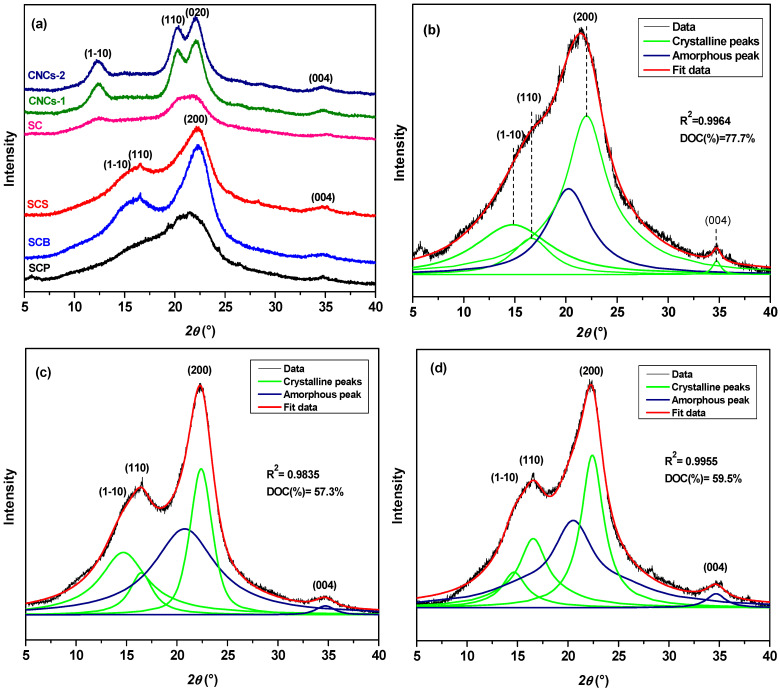

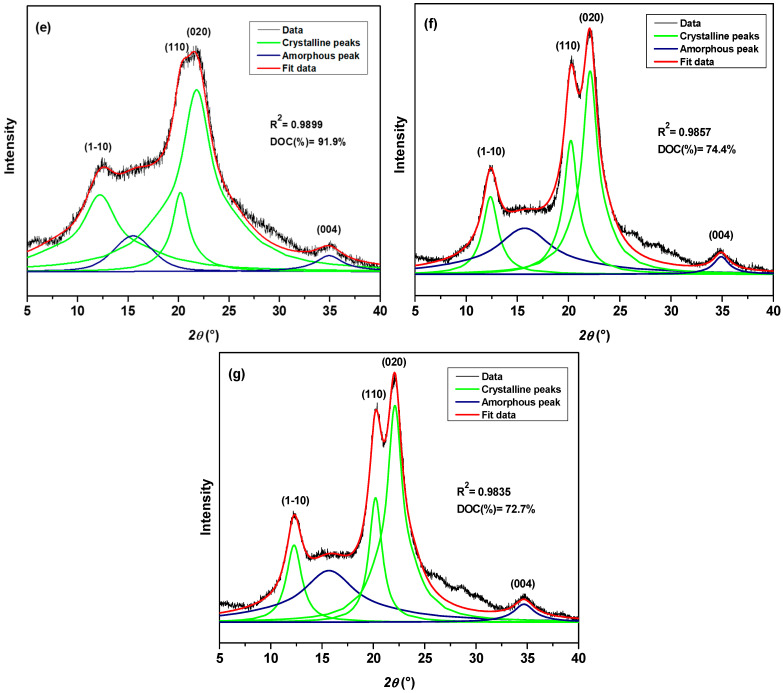
X-ray diffraction profiles during the treatment sequence: (**a**) all diffractograms, (**b**) SCP, (**c**) SCB, (**d**) SCS, (**e**) SC, (**f**) CNCs-1, (**g**) CNCs-2.

Figure 3 shows the X-ray diffractograms of SCP, SCB, SCS, CNCs-1, and CNCs-2, as well as the deconvolution of SCP and CNCs-1. The precursor sample visually presents an intense and broad peak, probably centered due to the overlap of crystalline reflections with the amorphous contribution, due to the presence of hemicellulose and lignin [65]. Peak deconvolution was therefore used (see Figure 3b), revealing the characteristic reflections of monoclinic cellulose I_β_, reflected in three main peaks with Miller indices of (1-10), (110), (200) and a secondary peak (004) corresponding to 2*θ* = 14.8°, 16.4°, 22.3°, and 34.6° [66]. On the other hand, a low-intensity peak was seen at 2*θ* = 20.2° for the amorphous region [57]. The SCB and SCS samples show similar X-ray diffractograms, and more defined peaks were observed due to the removal of hemicellulose and lignin after the application of NaCl_2_O and Na_2_SO_3_ [22,67,68]. After alkaline treatment, the peaks shifted from 2*θ* = 22.3° to 21.9° (020) and from 14.8° to 12.5° (1-10), reflecting the transition from polymorphs I to II [65]. It is suggested that this is due to the amount of Na^+^ ions available, which allows them to penetrate the crystalline regions, modifying the intermolecular hydrogen bonds of cellulose [69] and inducing structural alterations, reflected in a lower crystallinity index (see Table 2). The cellulose II polymorph has lower crystallinity but greater thermal stability. CNCs-1 and CNCs-2 showed diffraction peaks similar to those of SC, characteristic of the cellulose II polymorph, but they were more intense owing to the elimination of amorphous cellulose during H_2_SO_4_ hydrolysis, which was consistent with the results of ATR-FTIR spectroscopy.

**Figure 4:**
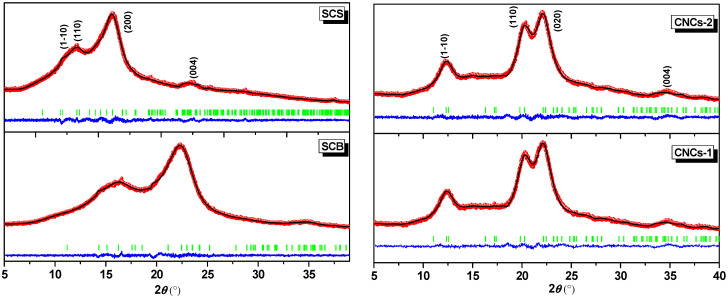
The Rietveld-refined X-ray diffractograms of the SCS, SCB, CNCs-1, and CNCs-2 samples. Experimental data are shown as red symbols, whereas the fitted profiles are displayed as black lines. Peak positions are indicated by green vertical lines, and the blue solid lines represent the differences between the experimental and refined curves. The *Y*-axis represents the intensity.

However, to truly confirm that these samples belonged to this polymorphic form of cellulose, Rietveld refinements were performed using the available crystallographic information. Figure 4 shows the X-ray diffractograms calculated from the crystalline models of cellulose I_β_ and cellulose II. It was verified that microcrystalline cellulose crystallized under a monoclinic crystal system, so it was identified as cellulose I_β_ for SCB and SCS, while, for CNCs-1 and CNCs-2, it was identified as the cellulose II polymorph, as shown in Table 3. The crystallite sizes identified for CNCs-1 (4.99 nm) and CNCs-2 (5.43 nm) were within the typical range reported for cellulose II obtained by acid hydrolysis. These crystallite dimensions are consistent with nanocrystalline cellulose domains obtained by acid hydrolysis and support the nanoscale nature of the obtained materials. Although morphological and surface analyses such as TEM, BET, or zeta potential analyses can provide complementary information, the present study was focused on crystallographic assignment, chemical purification, and process performance in COD removal. Consistently, Gong et al. [30] reported mean crystallite sizes of approximately 5.5 nm, which is close to that reported by Rana et al. [70] of 4.27–4.56 nm, ranging from ~4 to 6 nm, which are common even with variations in route/process. As Mahmud et al. [71] point out, changes in hydrolysis parameters (time, temperature) can alter the crystallinity and polymorphism of cellulose. In fact, as shown in Figure 4, variations in the H_2_SO_4_ concentration, temperature, and time resulted in slight variations in the average crystallite size for CNCs-1 and CNCs-2 [30,70,72]. Despite the close agreement of the refined unit cell parameters, confirming that both samples retained the cellulose II crystal structure, CNCs-1 and CNCs-2 differed mainly in lattice distortion, with CNCs-2 exhibiting a higher mean maximum strain. Since sulfuric acid hydrolysis preferentially attacks the amorphous regions [30], and its conditions govern the resulting CNC microstructure, the prolonged treatment used for CNCs-2 may have favored a more distorted cellulose II lattice than the shorter, more concentrated acid route used for CNCs-1 [73,74]. In contrast, the smaller SCS crystallite (3.73 nm) compared to SCB (5.03 nm) is consistent with a more aggressive pretreatment in terms of effective crystalline damage; the bleaching/delignification sequence may increase accessibility but also introduce crystalline defects and greater microstrain [44]. Specifically, the previous treatments (NaClO_2_/Na_2_SO_3_) preserved a crystalline fraction attributable to cellulose I_β_ in SCB/SCS, while the alkaline stage with 17.5% NaOH and subsequent hydrolysis favored reorganization into antiparallel chains and I → II conversion, consistent with what has been reported for mercerization in this NaOH range [75].

In this context, the combination of ATR-FTIR spectroscopy and XRD/Rietveld refinement is sufficient to support the structural assignment of cellulose II and the progressive removal of non-cellulosic components, which constituted the main characterization targets of the present work.

### 3.3. Box–Behnken Design

**Figure 5:**
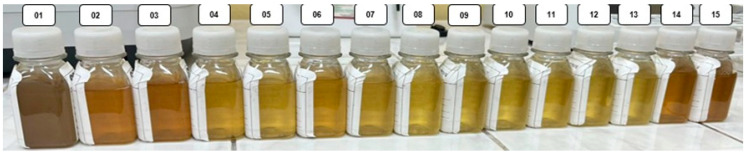
Results of the COD removal process: (1) MTD-01, (2) MTD-02, (3) MTD-03, (4) MTD-04, (5) MTD-05, (6) MTD-06, (7) MTD-07, (8) MTD-08, (9) MTD-09, (10) MTD-10, (11) MTD-11, (12) MTD-12, (13) MTD-13, (14) MTD-14, and (15) MTD-15.

Table 4 shows the results of the experiments carried out with the BBD, as well as the percentages of COD removal (see Figure 5). After selecting the appropriate model, a statistical analysis of the data was performed to evaluate their suitability. The results of the ANOVA for the polynomial response surface model are presented in Table 5. It can be observed that the probability values (*p*-value) are less than 0.05, indicating the significance of the applied model [76]. In addition, the mean squares of the lack of fit, which are not significant, indicate the suitability of the second-degree model and the absence of other relationships that affect removal efficiency.

The regression coefficient (R^2^) of 0.9675, close to the adjusted R^2^, implies that the model fits well and has acceptable predictive validity (predicted R^2^ = 0.8908) [46]. As for the adequate precision value of 16.0964, which is higher than 4, this shows that the signal-to-noise ratio is adequate, allowing the model to be used to predict COD removal under different experimental conditions [77]. Regarding the coefficient of variation (CV%), the value obtained of 0.5442 is less than 10, indicating the accuracy of the fit. An adequate approximation of the actual relationship between the response variable and the set of independent variables was obtained.

**Figure 6:**
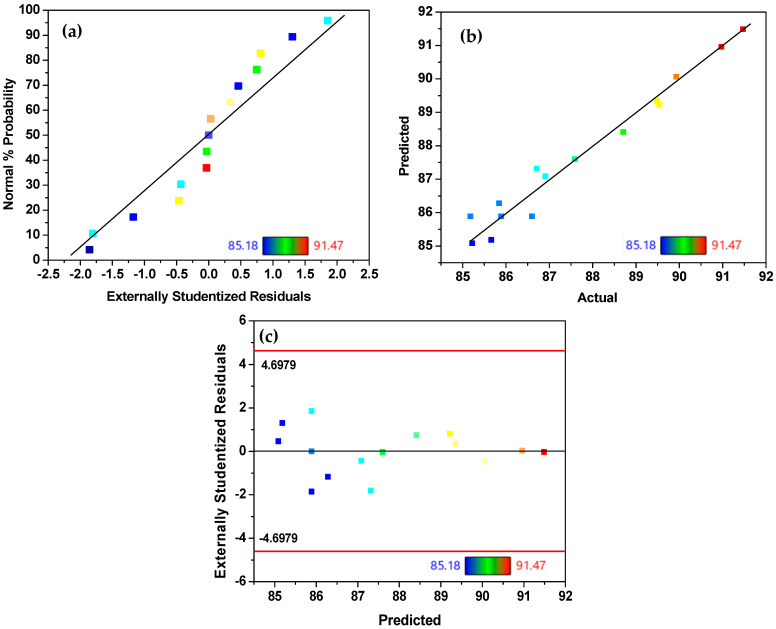
(**a**) Predicted vs. experimental results of removal efficiency in binary system, (**b**) normal probability plot of residuals, and (**c**) residuals vs. predicted; black line indicates the zero-residual reference, and red lines represent the critical limits for externally studentized residuals.

Once the suitability of the response surface polynomial model had been validated, a multiple regression analysis was performed using the Design-Expert software v13.0 to estimate the coefficients of the regression equation. This resulted in the formulation of a second-order polynomial equation (Equation (4)).

(4) $$D_{COD}\left(\%\right)=85.89-0.4037A-1.07B-2.09C+0.8525AC+0.9775A^{2}+0.8825B^{2}+1.56C^{2}$$

where D_COD_ (%) represents the percentage of COD removal, while A, B, and C refer to the CNCs-2 mass, contact time, and pH, respectively. The validity of the model was also evaluated by comparing the experimental and predicted responses of the experiments (see Figure 6). The normal probability plot evidences the normality of the residuals by maintaining a linear distribution (see Figure 6b) [78]. Likewise, the plot of observed versus predicted values shows that most data points are closely aligned with the 1:1 line, indicating good correspondence between them [79]. Additionally, the random distribution of the residuals around zero as a function of the predicted COD removal percentage confirms the adequacy of the model (see Figure 6c) [46]. The obtained results corroborate the statistical validity and the adequate predictive performance of the model.

These results support the suitability of the RSM as an optimization framework for COD removal from real industrial wastewater, where the response arises from a heterogeneous mixture of organic compounds rather than from the adsorption of a single, chemically defined solute.

### 3.4. Effect of CNCs-2 Mass

The effect of the CNCs-2 mass on the COD removal efficiency was evaluated in the range of 0.03–0.15 g. The highest performance was observed at the limits of the evaluated range, whereas a slight increase in the COD concentration occurred at 0.09 g, which can be attributed to system saturation and suggests an approach to equilibrium conditions [80]. The increase in COD removal efficiency may be associated with the higher availability of active sites on the CNCs-2 surface, while the decrease is likely related to overlap or aggregation, resulting in an increased diffusion path length [81].

Nevertheless, the statistical analysis indicates that the linear effect of the CNCs-2 mass on COD removal is not significant within the evaluated range (*p* = 0.0741), suggesting that the observed variations are not solely governed by mass but rather by the combined influence of other interactions within the system. Similar results were reported by Digal et al. [4], who applied nanocellulose derived from plant biomass for COD removal from dairy wastewater and achieved removal efficiencies of between 86 and 90.8% as the mass increased from 0.5 to 3 g. Similarly, Huaman and Peña [82] developed a chitosan–cellulose nanofibril aerogel (CNF/MPCS) using 0.12 and 0.3 g of each component, respectively, and reported COD removal efficiency of 45%.

### 3.5. Effects of Contact Time

An evaluation of the effects of the contact time on COD removal revealed that the removal efficiency increased with the contact time, reaching 91.47% after 20 min. This behavior can be attributed to the rapid initial interaction between the CNCs-2 and the COD, driven by the high availability of active sites on the structural surfaces of the CNCs-2 during the initial stage [83].

For contact times exceeding 20 min, the COD concentration approached a plateau with only minor variation, which can be associated with the saturation of adsorption sites and the attainment of the maximum adsorption capacity [84]. Therefore, extending the contact time did not result in a substantial improvement in COD removal. This behavior was also reported by Digal et al. [4], who identified an optimal contact time of 40 min, achieving COD removal efficiencies of approximately 86–88%, and noted that certain organic contaminants require longer times to diffuse into the internal pores of CNCs-2. Likewise, Huaman and Peña [82] reported a COD reduction of 41% at a contact time of 60 min.

### 3.6. Effects of pH

The influence of the pH on COD removal revealed a decrease in efficiency with increasing pH, with maximum removal of 91.47% achieved at pH 3. The COD in wastewater is primarily associated with organic compounds bearing functional groups such as carboxyl (–COOH) moieties [85]. Moreover, the surfaces of CNCs-2 contain hydroxyl (–OH) and sulfate (–OSO_3_^−^) groups [56].

Electrostatic interactions play a key role in the process. Abdelaziz et al. [86] reported a point of zero charge (pH_pzc_) of 5.4 for cellulose nanocrystal hydrogels. At pH values below those of the hydrogels, the CNCs-2 surface becomes positively charged due to the protonation of surface functional groups in the presence of excess H^+^ ions, leading to the formation of species such as (–OH_2_^+^) and (–OSO_3_H). This enhances electrostatic attraction with functional groups associated with COD. Conversely, at pH values above the pH_pzc_, the surface charge becomes increasingly negative, leading to reduced electrostatic attraction.

Moreover, a lower contribution from hydrogen bonding can be inferred due to the ionization of carboxylic functional groups, which typically exhibit pK_a_ values between 3.8 and 4.4 [87,88]. At pH 3, which is lower than the pK_a_, carboxyl groups remain predominantly protonated (–COOH), favoring intermolecular interactions with hydroxyl (–OH) groups on the CNCs-2 surface. At higher pH values, the deprotonation of these groups modifies the interaction mechanism by enhancing electrostatic effects.

This behavior is consistent with previous studies. Digal et al. [4] reported an optimal pH of 2 for achieving approximately 86% COD removal and observed decreasing removal efficiency with increasing pH in dairy wastewater. Likewise, Zhang et al. [83] reported improved COD removal at pH 4 using electrospun chitosan–cellulose acetate fibers, attributing this behavior to the protonation state of carboxylic groups in organic matter at pH values close to their pK_a_.

Moreover, relatively stable behavior is observed in the pH range of 5–7, which suggests the establishment of a balance among the interactions involved under the studied conditions. Notably, considerable COD removal is also achieved at a neutral pH (wastewater pH), minimizing the need for additional chemical reagents for pH adjustment. In line with this observation, Septevani et al. [35] applied activated carbon-functionalized nanocellulose to contaminated river water, achieving a 92.5% reduction in the initial COD concentration (1304.4 mg L^−1^) at pH 7.5.

To evaluate the novelty of this study, the results were compared with those reported in the literature for COD removal using different adsorbents, as summarized in Table 6. The CNCs-2 developed in this work achieved high COD removal efficiency at a relatively low dosage and short contact time, demonstrating their potential for treating palm oil plant wastewater. In addition, this approach promotes the valorization of empty fruit bunch residues from the same processing plant, converting an industrial byproduct into a value-added material for wastewater treatment.

### 3.7. Response Surface 3D Graph and Contour Plots of Interactive Effects

**Figure 7:**
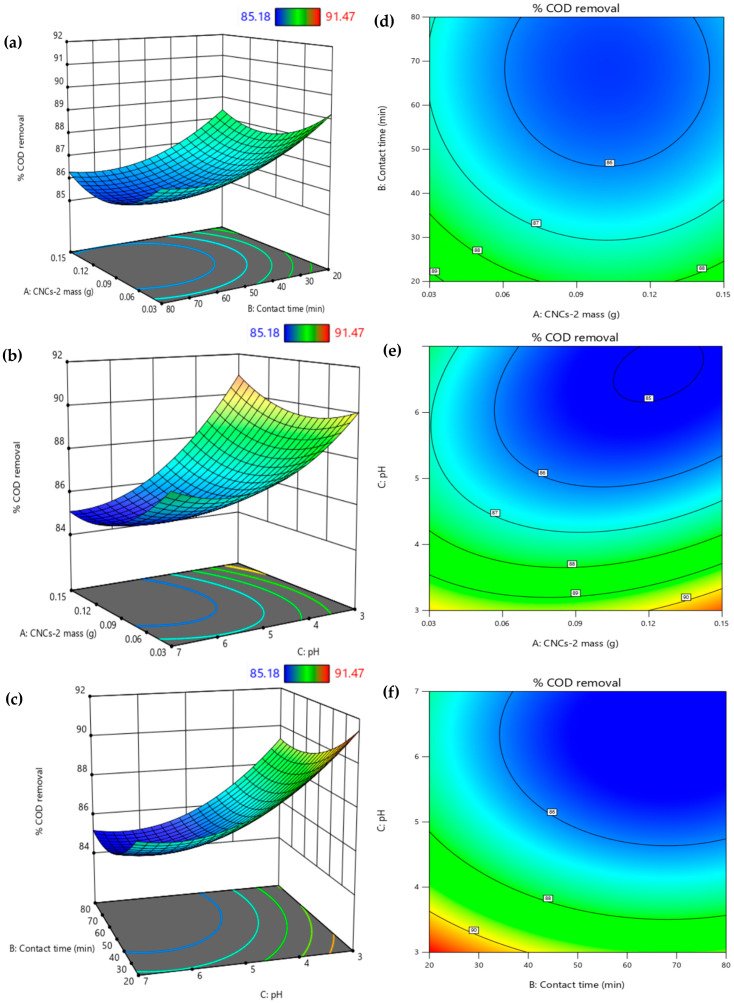
Three-dimensional response surface graphs and contour plots: (**a**) CNCs-2 mass vs. contact time; (**b**) CNCs-2 mass vs. pH; (**c**) contact time vs. pH; (**d**–**f**) corresponding contour plots. Colors represent COD removal: blue for low, green for intermediate, red for high.

Response surface plots and contour lines, based on the regression equation, allow for the optimization of process conditions and the visualization of factors affecting the system [46]. The results are shown in Figure 7. COD removal is greatest at low CNCs-2 concentrations and short contact times, with concentrations close to 87% (Figure 7a). The almost vertical orientation of the contour lines (Figure 7d–f) indicates that the contact time and pH have a greater influence than the CNCs-2 mass within the studied range. Increasing CNCs-2 does not significantly improve COD removal (Figure 7b), while a low pH and short contact time result in the maximum removal, indicated by the red region in Figure 7c. These results suggest that, once certain optimal conditions are reached, further variations in the variables do not produce significant improvements.

### 3.8. Possible Mechanism of Interaction

**Figure 8:**
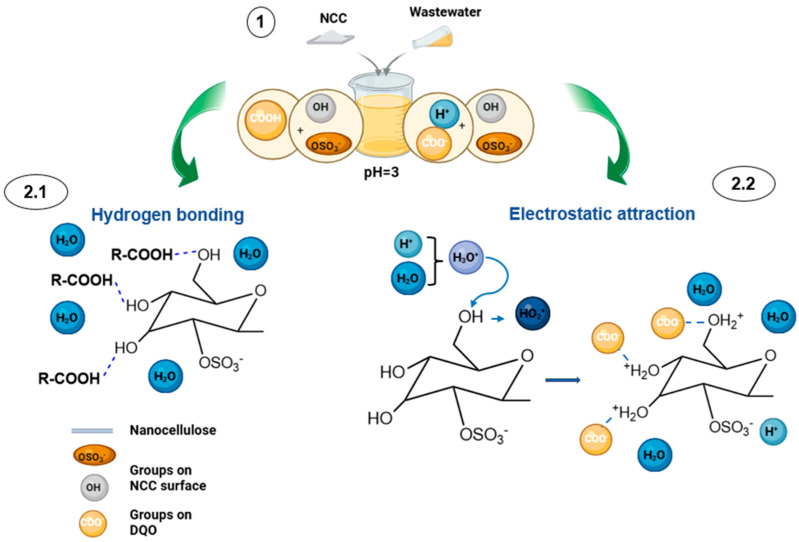
Schematic of possible electrostatic interaction on the surfaces of CNCs-2.

The mechanism proceeds through two main stages. In the first stage, hydrogen bond interactions dominate between hydroxyl groups on the CNCs-2 surface and oxygen-containing functional groups of the organic compounds contributing to the COD. At pH 3 (pH < pK_a_ 3.8–4.4) [87,88], carboxylic groups remain predominantly protonated (–COOH), favoring hydrogen bond formation with surface (–OH) groups. Additionally, these hydrogen bond interactions contribute to stabilizing the overall interfacial association (see Figure 8 (2.1)). In the second stage, electrostatic interactions become predominant and are controlled by the pH_pzc_, which reflects the acid–base equilibrium of surface functional groups. Ibrahim et al. [95] reported a pH_pzc_ of 6.5 for nanocellulose obtained via acid hydrolysis, whereas Abdelaziz et al. [86] reported a value of 5.4 for similarly hydrolyzed CNC. At pH 3, the condition yielding the best result is below these pH_pzc_ values (pH < pH_pzc_); the elevated H^+^ concentration promotes the protonation of surface (–OH) and (–OSO_3_^−^) groups, generating (–OH_2_^+^) and (–OSO_3_H) species and producing a net positive surface charge. This positively charged surface enhances electrostatic attraction with negatively charged species, particularly fatty acids present in their carboxylate form (–COO^−^) (see Figure 8 (2.2)).

## 4. Conclusions

Cellulose II nanocrystals were successfully synthesized from oil palm empty fruit bunch waste through sequential bleaching, delignification, mercerization, and controlled sulfuric acid hydrolysis. X-ray diffraction combined with Rietveld refinement confirmed the complete polymorphic transition from cellulose I_β_ to cellulose II, yielding CNCs with average crystallite sizes of 4.99 nm (CNCs-1) and 5.43 nm (CNCs-2). ATR-FTIR spectroscopy analysis corroborated the removal of lignin and hemicellulose and evidenced structural reorganization through characteristic hydroxyl and crystallinity-related band shifts. Among the synthesized materials, CNCs-2 exhibited superior adsorption performance and were selected for COD removal experiments. Using a Box–Behnken design, the COD removal process was statistically optimized, achieving maximum COD removal efficiency of 91.47% under optimal conditions (0.09 g, pH 3, and 20 min contact time). The quadratic response surface model demonstrated strong predictive capabilities, with R^2^ = 0.9675, adjusted R^2^ = 0.9349, and predicted R^2^ = 0.8908, confirming the robustness and reliability of the optimization. Process analysis revealed that the pH and contact time exerted a more significant influence on COD removal than the CNCs-2 mass within the studied range. The maximum removal was obtained under acidic conditions due to enhanced electrostatic interactions between protonated CNCs-2 surface groups (–OH_2_^+^, –OSO_3_H) and organic species contributing to the COD. Importantly, high COD removal efficiencies (>87%) were also achieved at a near-neutral pH, indicating the feasibility of treatment with reduced chemical adjustment. While the present work emphasizes structural confirmation and process optimization rather than exhaustive physicochemical profiling or classical single-solute adsorption modeling, the results clearly demonstrate the applicability of cellulose II nanocrystals for treating complex industrial wastewater. Overall, this study demonstrates that cellulose II nanocrystals derived from oil palm waste are a highly efficient, low-cost, and sustainable adsorbent for industrial wastewater treatment. The combination of high removal efficiency, a short contact time, and scalable synthesis highlights its strong potential for practical application in palm oil plant wastewater remediation and broader environmental engineering processes.

## Figures and Tables

**Table 1:** Experimental matrix for BBD.

Variables	Symbol	Unit	Range and Level		
			−1	0	+1
CNCs-2 mass	A	g	0.03	0.09	1.5
Contact time	B	min	20	50	80
pH of the treatment	C	-	3	5	7

**Table 2:** Calculated DOC values.

Sample	DOC (%)
SCP	77.7
SCB	57.3
SCS	59.5
SC	91.9
CNCs-1	74.4
CNCs-2	72.7

**Table 3:** Rietveld-refined parameters: lattices and volume cells, Caglioti parameters, and goodness of fit, with chi-square (χ^2^). *R*_p_ (%) is the profile refinement, *R*_wp_ (%) is the weighted profile residual, and *R*_exp_ (%) is the expected R-factor.

Refinement Parameter	SCB	SCS	CNCs-1	CNCs-2
a (Å)	8.216 (1)	8.107 (2)	7.926 (2)	7.945 (2)
b (Å)	6.084 (1)	6.314 (4)	9.025 (1)	9.042 (1)
c (Å)	9.916 (2)	10.447 (9)	7.409 (3)	7.399 (3)
α (°)	90.00 (0)	90.00 (0)	90.00 (0)	90.00 (0)
β (°)	90.00 (0)	90.00 (0)	90.00 (0)	90.00 (0)
γ (°)	105.47 (2)	99.58 (4)	117.32 (1)	117.43 (1)
$V\;({Å}^{3})$	477.68 (1)	527.25 (6)	470.82 (2)	471.82 (2)
FWHM parameters				
U	1.898	3.321	0.1766	0.2087
V	−0.7533	−0.2606	−0.6239	−0.6021
W	2.703	2.661	0.9584	0.9169
Microstructural parameters				
Mean maximum strain	79.38 (2)	98.42 (1)	36.96 (3)	72.69 (7)
Mean crystallite size (nm)	5.03 (5)	3.73 (7)	4.99 (2)	5.43 (2)
Statistical goodness-of-fit (GoF) parameters				
$R_{p}\;(\%)$	6.42	8.93	5.27	5.36
$R_{wp}\;(\%)$	6.26	8.65	5.66	5.72
$R_{exp}\;(\%)$	5.37	6.55	4.36	4.50
χ^2^	1.36	1.74	1.68	1.62

**Table 4:** BBD matrix.

Treatment	CNCs-2 Mass (g)	pH	Contact Time (min)	COD Removal				
				I	II	Average (mg L^−1^)	%	SD (±) *
MTD-01	0.15	7	50	369.9	357.2	351.55	85.22	12.7
MTD-02	0.09	7	80	345.5	359.7	376.60	85.66	14.2
MTD-03	0.09	5	50	326.4	328.7	327.55	86.68	2.3
MTD-04	0.15	5	20	271.1	284.2	277.65	88.71	13.1
MTD-05	0.03	5	80	326.4	317.6	322.00	86.91	8.8
MTD-06	0.15	5	80	353.7	342.9	348.30	85.84	10.8
MTD-07	0.09	3	80	263.7	253.4	258.55	89.49	10.3
MTD-08	0.09	5	50	317.1	318.3	317.75	86.15	1.3
MTD-09	0.03	3	50	235.6	259.6	247.6	89.93	24.0
MTD-10	0.03	5	20	256.5	258.1	257.30	89.54	1.6
MTD-11	0.09	3	20	214.0	205.7	209.85	91.47	8.3
MTD-12	0.15	3	50	220.7	223.6	222.15	90.97	2.9
MTD-13	0.09	5	50	312.0	319.3	315.65	87.16	7.3
MTD-14	0.03	7	50	311.0	299.1	305.05	87.59	11.9
MTD-15	0.09	7	20	332.1	321.3	326.70	86.71	10.8

* Standard deviation.

**Table 5:** ANOVA for COD removal.

Source	Sum of Squares	df	Mean Square	F-Value	*p*-Value
Model	61.64	6	8.81	29.73	0.0001
A	1.30	1	1.30	4.40	0.0741
B	9.10	1	9.10	30.71	0.0009
C	34.78	1	34.78	117.42	<0.0001
AC	2.91	1	2.91	9.81	0.0165
A^2^	3.53	1	3.53	11.91	0.0107
B^2^	2.88	1	2.88	9.71	0.0169
C^2^	8.99	1	8.99	30.34	0.0009
Residual	2.07	7	0.2962		
Lack of Fit	1.07	5	0.2130	0.4226	0.8108
Pure Error	1.01	2	0.5041		
Cor Total	63.71	14			
R^2^	0.9675		Std. Dev.	0.5442	
Adjusted R^2^	0.9349		Mean	87.71	
Predicted R^2^	0.8908		C.V.%	0.6205	
Adeq precision	16.0964				

**Table 6:** Comparison of COD removal efficiency with various adsorbents.

Adsorbent	$C_{0,COD}$ (mg L^−1^)	Dose (g L^−1^)	pH	Contact Time (min)	Treated Water	% Removal	Reference
Natural clay	1145.0	0.5	7.4	180	Textile effluent	66	[89]
Fe_3_O_4_-IDA-Cu^2+^ nanoparticles	-	1	4	60	Oilfield-produced water	66.7	[90]
TiO_2_ nanoparticles with chitosan	-	25	7.4	180	Petrochemical effluent	94.5	[91]
Activated carbon from avocado seeds	550.0	5	3	-	Petroleum refinery wastewater	94.54	[92]
CuO–activated carbon composite	-	1	7	60	Textile wastewater	72	[93]
Activated carbon	88.4	20	2	90	Synthetic wastewater	63.35	[94]
Modified carbon						75.41	
Nanocellulose (NCS05)	1304.4	-	7	-	River water	67.89	[35]
Nanocellulose acetate (NCA)	352.0	3	2	40	Synthetic wastewater	86	[4]
Cellulose nanocrystals (CNCs-2)	2459.0	0.5	3	20	Palm oil plant wastewater	91.47	(This study)

## Data Availability

The data presented in this study are available on request from the corresponding author.
